# Dr. Harry Mead

**Published:** 1917-04

**Authors:** 


**Dr. Harry Mead,** Buffalo 1891, died suddenly at his home, 758 Elmwood Ave., Buffalo, Mch. 18, aged 47. He was born in Youngsville, Pa., July 11, 1869, came to Buffalo as a boy and attended the Central (now Hutchinson) High School, after graduating from which, he entered the University of Buffalo. He studied after graduation, in Germany, France and Austria. He was affiliated with the Buffalo Academy of Medicine and the A. M. A. and its state and county societies. He was a member of the faculty of the Medical Dept. of the University of Buffalo and of the staff of the Buffalo General Hospital. He was, at the time of his death, Secretary of the Medical Section of the Academy. A more extended memorial will be published later.

